# EXPLORING STRUCTURAL BARRIERS TO MEDICAL CANNABIS ACCESS AMONG OLDER ADULTS

**DOI:** 10.1093/geroni/igz038.736

**Published:** 2019-11-08

**Authors:** Kanika Arora, Kanika Arora, Julie Bobitt, Brian Kaskie

**Affiliations:** 1 University of Iowa, College of Public Health, Iowa City, Iowa, United States; 2 University of Iowa, Iowa City, Iowa, United States; 3 University of Illinois at Urbana Champaign, Champaign, Illinois, United States

## Abstract

A growing number of older persons are using cannabis to treat medical conditions and symptoms. Preliminary work examining survey responses from persons over age 60 in Colorado and revealed the presence of structural barriers in safe and effective access to MC. In particular, older adults reported a gap in their expectations as a patient and current healthcare provider practice in counseling and educating them on MC use. To examine this from the provider perspective, we aim to survey Illinois state physicians and assess current knowledge, training needs, attitudes and practices associated with MC and older adults. Multivariate regression analysis will be conducted to predict current practice associated with MC (for example, whether physician ever certified a patient for MC) as a function of provider characteristics, as well as their knowledge and attitudes on the topic. We will also conduct sub-group analysis to understand practice patterns by specialty and experience.

